# Rethinking the Neural Basis of Prosody and Non-literal Language: Spared Pragmatics and Cognitive Compensation in a Bilingual With Extensive Right-Hemisphere Damage

**DOI:** 10.3389/fpsyg.2019.00570

**Published:** 2019-03-19

**Authors:** Noelia Calvo, Sofía Abrevaya, Macarena Martínez Cuitiño, Brenda Steeb, Dolores Zamora, Lucas Sedeño, Agustín Ibáñez, Adolfo M. García

**Affiliations:** ^1^Laboratory of Experimental Psychology and Neuroscience, Institute of Cognitive and Translational Neuroscience, INECO Foundation, Favaloro University, Buenos Aires, Argentina; ^2^National Scientific and Technical Research Council, Buenos Aires, Argentina; ^3^Faculty of Psychology, National University of Córdoba, Córdoba, Argentina; ^4^Laboratory of Language Research (LILEN), Institute of Cognitive and Translational Neuroscience (INCYT), Buenos Aires, Argentina; ^5^Universidad Autónoma del Caribe, Barranquilla, Colombia; ^6^Department of Psychology, Universidad Adolfo Ibáñez, Santiago, Chile; ^7^Centre of Excellence in Cognition and Its Disorders, Australian Research Council, Sydney, NSW, Australia; ^8^Faculty of Education, National University of Cuyo, Mendoza, Argentina

**Keywords:** pragmatics, bilingualism, adult-onset stroke, right hemisphere lesions, cognitive compensation

## Abstract

Above and beyond the critical contributions of left perisylvian regions to language, the neural networks supporting pragmatic aspects of verbal communication in native and non-native languages (L1s and L2, respectively) have often been ascribed to the right hemisphere (RH). However, several reports have shown that left-hemisphere activity associated with pragmatic domains (e.g., prosody, indirect speech, figurative language) is comparable to or even greater than that observed in the RH, challenging the proposed putative role of the latter for relevant domains. Against this background, we report on an adult bilingual patient showing preservation of pragmatic verbal skills in both languages (L1: Spanish, L2: English) despite bilateral damage mainly focused on the RH. After two strokes, the patient sustained lesions in several regions previously implicated in pragmatic functions (vast portions of the right fronto-insulo-temporal cortices, the bilateral amygdalae and insular cortices, and the left putamen). Yet, comparison of linguistic and pragmatic skills with matched controls revealed spared performance on multiple relevant tasks in both her L1 and L2. Despite mild difficulties in some aspects of L2 prosody, she showed no deficits in comprehending metaphors and idioms, or understanding indirect speech acts in either language. Basic verbal skills were also preserved in both languages, including verbal auditory discrimination, repetition of words and pseudo-words, cognate processing, grammaticality judgments, equivalent recognition, and word and sentence translation. Taken together, the evidence shows that multiple functions of verbal communication can be widely spared despite extensive damage to the RH, and that claims for a putative relation between pragmatics and the RH may have been overemphasized in the monolingual and bilingual literature. We further discuss the case in light of previous reports of pragmatic and linguistic deficits following brain lesions and address its relation to cognitive compensation in bilingual patients.

## Introduction

In addition to central linguistic functions (phonology, lexical semantics, and syntax), verbal communication in both native and non-native languages (L1s and L2s, respectively) is crucially rooted in pragmatic domains. The latter comprise diverse abilities that allow people to exchange meanings beyond the literal form of an utterance (Searle et al., 1980; Tompkins, 1995; Sperber and Wilson, 2005; Stemmer, 2008) and to evaluate whether a piece of discourse is meant as a question, an indirect request, or a figurative construction (Levinson, 1983), among others. This implies different inferential and comprehension processes operating at the supra-sentential level, such as understanding the illocutionary force of a statement, integrating its meaning with contextual information, and deriving another interpretation if the literal one is found to be inappropriate (Giora, 1999; Stemmer, 2008). The processes involved also draw on prosodic features (intonation, boundary tones, pausing, pitch accents, melody contour), which are critical to resolve semantic ambiguities and guide the listener’s interpretation (Grice, 1975; Searle, 1979; Ortony, 1993; Gibbs, 1994; Wilson and Wharton, 2006).

Traditionally, these and other pragmatic abilities have been proposed to rely predominantly on the right hemisphere (RH) (Joanette et al., 1990; McDonald, 1999), a claim that has also been explicitly postulated in models of bilingual processing (Paradis, 2009). Yet, while such a position aligns with reports of impaired verbal pragmatics following RH lesions (Bryan, 1989; Kaplan et al., 1990; Lindell, 2006; Champagne-Lavau and Joanette, 2009), it is challenged by other strands of evidence. For example, several studies have shown pervasive left-hemisphere (LH) contributions to pragmatic domains, including processing of figurative language (Bloom et al., 1993; Rapp et al., 2004; Uchiyama et al., 2012) and indirect speech acts (Soroker et al., 2005). The same is true of prosodic skills: while some studies have emphasized the role of the RH in both linguistic (Weintraub et al., 1981; Brådvik et al., 1991) and emotional (Blonder et al., 1991; Starkstein et al., 1994; Ross and Monnot, 2008) dimensions, others have linked such domains predominantly to the LH (Van Lancker, 1980; Emmorey, 1987; Brådvik et al., 1991; Baum et al., 1997; Gandour et al., 2004).

Also, pragmatic processing may be partially subserved by the prefrontal cortex, which coordinates multiple neural networks mediating cognitive control and social functioning (Cummings, 1993; Fuster, 2001; Stemmer, 2008; Stemmer and Whitaker, 2008; Anderson et al., 2010). Of note, these regions exhibit functional and structural changes in bilinguals (Abutalebi et al., 2001; Mechelli et al., 2004; Pliatsikas et al., 2015), who typically outperform monolinguals in their executive performance (Bialystok et al., 2009; Bialystok and Craik, 2010) and their cognitive outcomes after stroke (Alladi et al., 2015; Paplikar et al., 2018). Therefore, spared prefrontal functioning in bilinguals, arguably due to cognitive compensation, could also account for preserved pragmatic performance even despite RH lesions.

Taken together, this evidence challenges straightforward conceptualizations of the putative role of the RH in pragmatic aspects of verbal communication, suggesting that such a relation may have been overemphasized in the literature. Here, we examine the issue by assessing pragmatic and linguistic skills in a cognitively preserved bilingual patient exhibiting extensive, adult-onset, cortical and subcortical RH lesions, and less profuse LH damage (García et al., 2017).

If the putative neural substrates subserving pragmatic functions were predominantly rooted in the RH, then the patient should evince profuse deficits in relevant tasks. Strikingly, however, despite mild deficits in L2 prosodic skills, the patient exhibited widespread sparing of prosody in L1 and figurative language in both languages, alongside well-preserved lexical, sentence-level, and cross-linguistic skills. Accordingly, this case invites new reflections on the neurobiological basis of pragmatic functions, indicating that these may be less dependent on RH integrity than proposed in previous works and informing their potential relation with cognitive compensation in bilingual patients.

## Materials and Methods

### Case Report

CG is a 46-year-old, right-handed Argentinean woman with 18 years of formal education and high proficiency in Spanish (L1) and English (L2). She reported no neurological or psychiatric antecedents, and no history of familial sinistrality. She first became exposed to English at the age of two and attended a bilingual school for 7 years, where she took all subjects in Spanish and English. Later, she took private English lessons for 9 years and traveled to different countries where she mainly spoke this language. Also, during her appointment as a financial executive at an international bank in Argentina, she used her L2 daily in oral and written communications. Even after her two strokes, she reported being able to understand complex L2 materials, such as full scientific conferences.

**FIGURE 1:**
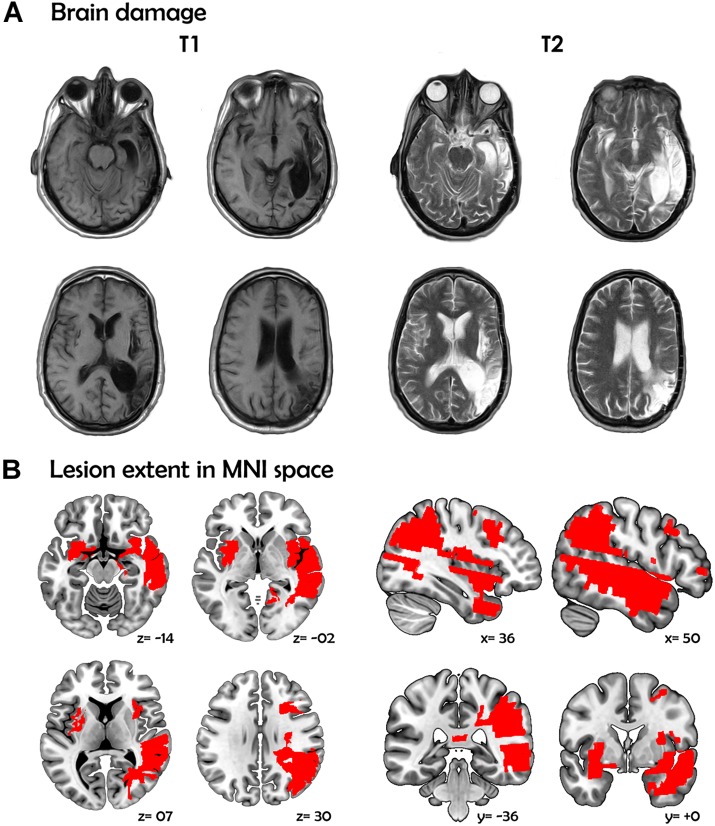
MRI scans and lesion extent of patient CG. **(A)** Brain damage. T1 and T2 image showing axial views of the patient’s brain. **(B)** Lesion extent in MNI space. Multislice overlap of lesions within a normalized brain from the MNI brain atlas. On the right hemisphere, these included the fronto-insulo-temporal cortices, spanning from the medial anterior temporal lobe (parahippocampal gyrus and amygdala) to the mid and superior temporal gyri; the supramarginal and angular gyri; the inferior parietal lobule; almost the complete insula; and a portion of the putamen and the inferior frontal operculum. On the left hemisphere, compromised regions included the left anterior insula and its underlying white matter, the putamen, and the dorso-lateral amygdala. All images are in neurological orientation.

On September 9, 2011, at the age of 43, CG suffered from sudden severe headache, nausea, and loss of consciousness. During hospitalization, radiological findings revealed a subarachnoid hemorrhage (Fisher scale: grade IV; Hunt-Hess scale: grade V) due to a ruptured 5-mm fusiform aneurysm at the right medial cerebral artery which later complicated with severe vasospasm, leading to extensive damage affecting multiple RH regions, namely: the medial anterior temporal lobe (parahippocampal gyrus and amygdala), the mid and superior temporal gyri, the supramarginal and angular gyri, the inferior parietal lobule, the complete insula, a portion of the putamen, and the inferior frontal operculum (Figure 1; for details on the extension of the lesion in each area and additional images highlighting the involvement of the left and right amygdala, see Supplementary Material). Of note, such regions have been previously related to inferencing (Martin and McDonald, 2005, 2006), prosody production (Blumstein and Cooper, 1974; Bryan, 1989; Dykstra et al., 1995), and non-literal language comprehension (Martin and McDonald, 2005; Côté et al., 2007; Martín-Rodríguez and León-Carrión, 2010). She spent 41 days in intensive care and was then discharged with moderate left-sided hemiparesis.

Two years later, a second (ischemic) stroke produced by a sudden reswallowing (presumably related to a previous craniotomy) induced additional damage in the LH, including the anterior insula and its underlying white matter, the putamen, and the dorso-lateral amygdala (Figure 1). These regions have been implicated in bilingual processing via phonological (Klein et al., 1994; Chee et al., 2004; Tettamanti et al., 2005; Abutalebi et al., 2013), semantic (Copland et al., 2002; Wartenburger et al., 2003) and syntactic (Tschirren et al., 2011; Park et al., 2012; Hernandez et al., 2015) tasks.

Exceptionally, after clinical stabilization, CG did not show noticeable neurological, cognitive, emotional or behavioral impairments. She only reported loss of sensitivity on the right hand and a transient form of personality-color synesthesia (Ramachandran et al., 2012), which were resolved after therapy. No further signs of focal neurological deficits were reported.

Formal neuropsychological evaluations after discharge (all performed in her L1) revealed high preservation of executive functions –scoring 25/30 on the INECO Frontal Screening (IFS) battery (Torralva et al., 2009)– and overall cognitive status –with a score of 96/100 on the Argentine adaptation of the Addenbrooke’s Cognitive Examination Revised (ACE-R) (Torralva et al., 2011). Comparisons of these outcomes with sociodemographically matched controls –for details, see García et al. (2017)– showed no significant differences in the IFS (Crawford’s *t*-test = 0.07, *p* = 0.94) or in the ACE-R (Crawford’s *t*-test = -1.17, *p* = 0.29).

### Control Sample

The patient’s performance on linguistic and pragmatic tasks was compared with that of eight healthy bilingual women reporting no history of neurological or psychiatric disease. All controls were self-identified as highly competent speakers of Spanish (L1) and English (L2). As shown through Crawford’s modified *t*-tests (Crawford and Howell, 1998; Crawford et al., 2009, 2010), this sample was matched with CG in terms of age (Crawford’s *t*-test = -0.19, *p* = 0.852), years of education in L1 (Crawford’s *t*-test = -0.31, *p* = 0.764), and years of education in L2 (Crawford’s *t*-test = 0.19, *p* = 0.849). Note, however, that CG had a significantly earlier age of L2 acquisition (Crawford’s *t*-test = 3.15, *p* = 0.016). For more details, see Table 1.

**Table 1:** Demographic data from the patient and controls.

	Patient	Controls (*n* = 8)	*SD*	*t*-Value	*z_cc_*	*p*-value
Age	46	47	4.86	-0.19	-0.20	0.85
Age of L2 acquisition	2	10	2	-3.15	-3.34	0.01
Years of education in L1	18	19	3.02	-0.31	-0.33	0.76
Years of education in L2	16	14	10	0.19	0.20	0.84


This study was carried out in accordance with the recommendations of the Ethics Committee of the Institute of Cognitive Neurology (INECO, now a host institution of the Institute of Cognitive and Translational Neuroscience). All subjects gave written informed consent in accordance with the Declaration of Helsinki, and the patient also provided written informed consent for the publication of this case report. The protocol was approved by the Ethics Committee of INECO. All data analyzed in the study are available upon request.

### Assessment of Overall Linguistic Profile

#### Language History and Basic Linguistic Skills

As linguistic variability is expected when assessing bilingualism (for a review, see Calvo et al., 2016a), basic verbal skills in and between L1 and L2 were assessed to determine a linguistic profile for each subject in each language. In the first part, we used the Spanish version of the Language History Questionnaire 2.0 (LHQ 2.0) (Li et al., 2014) and part A from the Spanish-English version (Muñoz and Marquardt, 2008) of the Bilingual Aphasia Test (BAT) (Paradis, 1987), a validated tool designed to examine all levels of linguistic structure in the four modalities of language in bilinguals with diverse neurological and developmental disorders (Paradis, 2011).

Moreover, our protocol included two subsets of tasks tapping lexical and sentence-processing abilities. L1- and L2-specific tasks were selected from part B of the short versions of the American-Spanish and the English versions of the BAT, whereas the translation tasks corresponded to part C. Each task was identical between languages in terms of structure, number of items, administration, and scoring criteria. In each task, the examiner addressed the participant exclusively in the language being assessed. All tasks were administered following published instructions (Paradis, 1987; Paradis and Libben, 2014). Taken together, this part of protocol lasted roughly 2 h per participant.

#### Word-Level Tasks

Word-level skills in each language were assessed through four tasks from part B of the BAT (Paradis, 1987), chosen to cover key sublexical, lexical, and semantic abilities. Then, cross-linguistic lexical processing was evaluated through two tasks from part C, tapping on translation equivalent recognition and word translation skills.

##### Naming

Twenty ordinary objects were shown by the examiner (e.g., a book, a box of matches, a fork, a candle) and the participant had to name them. Each correct answer received a score (maximum score: 20/20 in each language).

##### Verbal auditory discrimination

A picture was shown alongside a target word and three rhyming foils, and the subject had to finger-point in response to a verbal presentation of the target word read by the examiner. Eighteen stimuli were presented and the subject scored one point for each target word identified (maximum score: 18 for each language).

##### Word comprehension

Word comprehension was assessed with a silent reading task in which participants had to identify a figure given an orally presented word. Here, 10 words were read and each correct response received one point (maximum score: 10 for each language).

##### Word repetition

Participants had to repeat a word uttered by the examiner and then decide if that was a real word in the language being assessed. Thirty stimuli (20 nouns and 10 pseudowords) were presented in each language, (maximum score: 30 in each language).

##### Equivalent recognition

Participants were shown a list of words in one language and they had to identify their equivalent in the other language. Five words were presented in their L1 and five in their L2. A score was given for each correct answer (maximum score: 5 in each language).

##### Word translation

Subjects were read 10 words in each language and they had to translate them in the corresponding direction (Spanish-English/English-Spanish) (e.g., *cuchillo-knife, fork-tenedor*). Each correct response received one point (maximum score: 10 for each language).

#### Sentence-Level Tasks

Similarly, sentence processing skills in each language were assessed through three tasks per language from part B of the BAT (Paradis, 1987), while cross-linguistic sentence processing was examined with a relevant translation task from part C of the same instrument.

##### Grammatical correction

Subjects were asked to correct sentences from the previous task in the language being assessed (e.g., *She went to work without eating breakfast*). Two scores were allotted for each sentence (with a maximum of 8 for judgment and 8 for correction in each language).

##### Grammaticality judgment

Participants first read eight sentences (a mix of declarative, affirmative, negative, and interrogative constructions) and decided whether each sentence was correctly formed. Incorrect sentences contained typical mistakes in prepositions, infinitives and gerunds (e.g., *She went to work without to eat breakfast*).

##### Sentence comprehension

This domain was evaluated through a silent reading task. Participants were shown different pictures together with written sentences and they had to identify the picture that best described the meaning of that sentence (e.g., “*the dog is bitten by the cat*,” “*he holds the girl*”). A set containing 10 affirmative and negative sentences in active or passive voice was presented and each correct sentence received one point (maximum score: 10 for each language).

##### Sentence translation

Six affirmative sentences were read aloud in each language and the participant had to translate them in each direction (Spanish-English/English-Spanish) (e.g., *Mi amigo ha trabajado en Miami durante dos meses/My friend has worked in Miami for 2 months*). Sentences were read to the participant up to three times in accordance with his/her request for repetition and the score corresponded to the number of times that the text was read and the number of word groups containing no errors. Thus, a total of three points were given per correct sentence (maximum score: 18 for each language).

### Assessment of Pragmatic Functions

Pragmatic functions in L1 and L2 were assessed through the Montreal Evaluation of Communication (MEC) (Ferreres et al., 2007) and the Pragmatic English Assessment for Spanish Speakers (PEASS) (Zamora and Steeb, 2016), respectively. The MEC is a normalized clinical tool for assessing pragmatic and communicative skills in Spanish-speaking patients. This test taps several crucial domains of functional language (figurative language, prosody production) and it has been used to examine pragmatic functions after RH damage in previous studies (Joanette et al., 2008; Tabernero and Politis, 2012). The PEASS is an English version of the MEC, which was specially designed for this evaluation by specialists in the subject at hand. Its principal aim is to assess English (L2) pragmatic-communicative functions in native Spanish speakers, via stimuli that consider cross-linguistic particularities of the English-Spanish language pair. All the tasks in this part of protocol were identical between languages in terms of structure, number of terms, administration, and scoring criteria. Administration of both tests lasted approximately 90 min per participant.

#### Non-literal Communication Tasks

##### Idioms

Participants were presented with 10 sentences containing idiomatic expressions (e.g., *She’s biting more than she can chew)* and they were asked to choose its correct meaning from three options [e.g., (a) *she’s eating a lot*; (b) *she always puts too much food in her mouth*; (c) *she is trying to do more than what she is able to do*]. The scoring was the same as the previous task, with one point given for each correct sentence (maximum score: 10 in each language).

##### Indirect speech acts

Inferencing of implicit meanings was assessed through a task containing indirect speech acts. Twenty situations (in each language) were presented in form of a short text that the examiner read to the participants (e.g., *Adrian is waiting for his girlfriend at the cinema because they are going to see a movie. As always, she arrives late so when they meet, he asks: “Did you get lost?*”). Then, the examiner presented two options [e.g., (a) *Adrian wants to ask her if she had problems finding the way to the cinema*; (b) *Adrian wants to point out she was late*] and the participant had to choose which option explained the sentence better. Ten of these situations had an implied meaning and a point was given for each correct sentence (maximum score: 10 for implied meanings in each language).

##### Metaphors

Participants read 10 metaphors (e.g., *Rebecca, your house is the North Pole)*, they were presented with three options and had to choose the one that explained the sentence’s meaning better [e.g., (a) *Rebecca’s house is really cold*; (b) *Rebecca lives in the North Pole*; (c) *Rebecca’s house is full of snow*]. One point was given for each correct sentence (maximum score: 10 in each language).

#### Prosodic Tasks

##### Emotional prosody comprehension

Subjects listened to 12 recorded sentences which varied in intonation and pitch levels and had to recognize the speaker’s emotions (happy, sad or angry). The answer was measured as right or wrong and the maximum score was 12 in each language.

##### Emotional prosody production

Production was evaluated with a task in which participants were visually presented a sentence, then they were read a short text and they were asked to say the sentence they first read using an intonation that matched the content of the short text. Here, three sentences were presented and three different texts were read for each sentence, so that the participant said each sentence using the three emotions (sad, angry, happy). Each correct answer received a score and thus the maximum score was 18 for each language.

##### Emotional prosody repetition

Participants listened again to the 12 sentences already introduced in the first task and repeated them respecting the correct intonation. Answers were rated as right or wrong and the maximum score was 12 in each language.

##### Linguistic prosody comprehension

This test comprised 12 short recorded sentences, which were uttered as a statement, a question or an exclamatory sentence. The participant listened to the recorded sentences (in sum four of each type) and had to recognize the intonation used. The answer was measured as right or wrong and one point was given for each correct sentence (maximum score: 12 in each language).

##### Linguistic prosody repetition

The same 12 stimuli used in the previous task were given again and the participant had to repeat each sentence with the same pitch he/she heard and the maximum score was 12 in each language.

### Statistical Analysis

The patient’s demographic, neuropsychological, and experimental data were compared to those of the control sample via Crawford’s modified two-tailed *t*-test (Crawford and Howell, 1998). This test is widely used for non-normal distributions, presents low rates of Type-I error, and has proved successful in previous single-case studies (Baez et al., 2013; Sedeño et al., 2014; García et al., 2017), even when the control sample comprises fewer than five subjects (Straube et al., 2010). Given that the patient had a significantly lower AoA than the controls, all statistical analyses were performed with such a factor as a covariate to rule out its potential influence on the results. Alpha levels were set at *p* < 0.05 and effect sizes (*z_cc_*) for differences between case and controls were obtained with point estimates, as suggested in the literature (Crawford et al., 2010).

## Results

### Basic Bilingual Skills

#### Performance on Word-Level Tasks

**FIGURE 2:**
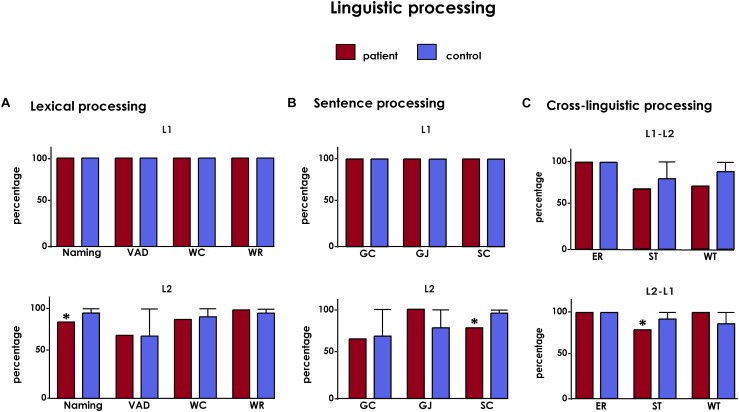
Results from basic bilingual tasks. **(A)** Lexical processing: VAD, verbal auditory discrimination; WC, word comprehension; WR, word repetition. **(B)** Sentence processing: GC, grammatical correction; GJ, grammatical judgment; SC, sentence comprehension. **(C)** Cross-linguistic processing: ER, equivalent recognition; WT, word translation; ST, sentence translation. All results are shown after covariation with AoA and are presented in percentage values. Asterisks (^∗^) indicate statistical differences at *p* < 0.05.

CG exhibited ceiling-level performance in all tasks tapping on lexical processing in her L1 (Figure 2A). Accordingly, no statistical tests were run for auditory discrimination, word repetition, naming, and word comprehension tasks in this language.

The patient’s lexical skills were also widely spared in L2. Her performance was optimal on word repetition, as was that of controls (therefore, no statistical analysis was necessary). She also exhibited no deficits on verbal auditory discrimination (Crawford’s *t*-test = 0.17, *p* = 0.440, *z_cc_* = -1.47) or word comprehension (Crawford’s *t*-test = -0.79, *p* = 0.114, zcc = -3.29). Also, she achieved a high numerical score on word naming (18/20), although the two mistakes she made resulted in statistical differences relative to controls (Crawford’s *t*-test = -5, *p* = 0.015, *z_cc_* = -5.95) (Figure 2A).

Finally, the patient’s cross-linguistic lexical processing skills were also well preserved. CG, as well as every other participant, obtained perfect scores on both equivalent recognition tests (L1–L2 and L2–L1) –therefore, no statistical analysis was run. Also, her word translation skills revealed a slight trend toward significance in L1–L2 (Crawford’s *t*-test = -1.82, *p* = 0.054, *z_cc_* = -4.25) but no deficit in the L2–L1 direction (Crawford’s *t*-test = 0.50, *p* = 0.466, *z_cc_* = -1.38) (Figure 2C).

Taken together, these results show that CG’s basic lexical processing skills were largely spared in both languages.

#### Performance on Sentence-Level Tasks

Both CG and the control group obtained maximum scores in grammaticality judgments and grammatical corrections in L1. Neither did the patient exhibit difficulties in sentence comprehension (Crawford’s *t*-test = 0.50, *p* = 0.279, *z_cc_* = -2.12) (Figure 2B).

Results from L2 tasks showed preserved performance on grammatical judgments (Crawford’s *t*-test = 1.01, *p* = 0.932, *z_cc_* = 0.15) and grammatical corrections (Crawford’s *t*-test = -0.32, *p* = 0.290, *z_cc_* = -2.06). This was accompanied by a considerably high score (8/10) in sentence comprehension, which nonetheless differed significantly from the near-ceiling performance of controls (Crawford’s *t*-test = -5, *p* = 0.003, *z_cc_* = -8.12) (Figure 2B).

Finally, the patient evinced no impairments in translating sentences from L1 into L2 (Crawford’s *t*-test = -1.63, *p* = 0.175, *z_cc_* = -2.74) but statistical differences were shown from L2 into L1 (Crawford’s *t*-test = -2.05, *p* = 0.019, *z_cc_* = -5.65) (Figure 2C).

Taken together, these results indicate that CG’s sentence processing skills were widely preserved.

### Pragmatic Functions

#### Performance on Non-literal Communication Tasks

**FIGURE 3:**
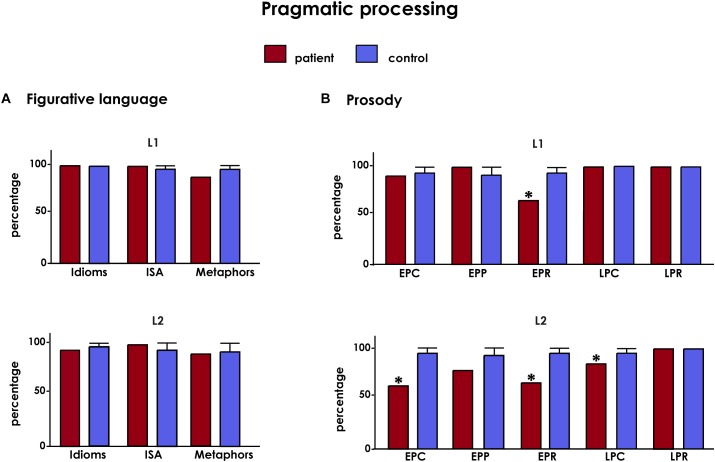
Results from pragmatic processing tasks. **(A)** Figurative language: ISA, indirect speech acts. **(B)** Prosody: EPC, emotional prosody comprehension; EPP, emotional prosody production; EPR, emotional prosody repetition; LPC, linguistic prosody comprehension; LPR, linguistic prosody repetition. All results are shown after covariation with AoA and are presented in percentage values. Asterisks (^∗^) indicate statistical differences at *p* < 0.05.

In general, CG’s performance on non-literal comprehension tasks was similar to that of controls across all domains assessed, both in L1 (indirect speech acts: Crawford’s *t*-test = 0.33, *p* = 0.662, *z_cc_* = -0.82; metaphors: Crawford’s *t*-test = -2.33, *p* = 0.134, *z_cc_* = -3.08; idioms: all subjects obtained perfect scores) and in L2 (indirect speech acts: Crawford’s *t*-test = 0.33, *p* = 0.908, *z_cc_* = -0.21; metaphors: Crawford’s *t*-test = -0.10, *p* = 0.379, *z_cc_* = -1.69; idioms: Crawford’s *t*-test = -2.33, *p* = 0.134, *z_cc_* = -3.08). Taken together, these results show that the patient’s figurative language processing skills were nearly fully spared in both L1 and L2 (Figure 3A).

#### Performance on Prosodic Tasks

Results from linguistic prosody showed maximum scores in the repetition and comprehension task for all participants in L1. The scoring for repetition in L2 was also perfect for all participants, but CG’s two mistakes in the comprehension task (10/12) yielded a statistically significant difference (Crawford’s *t*-test = -5, *p* = 0.015, *z_cc_* = -5.95).

No differences were found in emotional prosody comprehension (Crawford’s *t*-test = -2.33, *p* = 0.134, *z_cc_* = -2.12) or production in L1 (Crawford’s *t*-test = 0.44, *p* = 0.908, *z_cc_* = -0.21). However, emotional prosody in L1 yielded differences for repetition relative to controls (Crawford’s *t*-test = -10.33, *p* < 0.001, *z_cc_* = -11.69).

Finally, CG showed impairments in emotional prosody in L2, for repetition (Crawford’s *t*-test = -10.33, *p* = *p* < 0.001, *z_cc_* = -11.69) and comprehension (Crawford’s *t*-test = -13, *p* < 0.001, *z_cc_* = -14.56). Emotional prosody production showed only marginal differences (Crawford’s *t*-test = -3, *p* = 0.071, *z_cc_* = -3.80). Interestingly, though, all her mistakes in emotional prosody (for repetition in L1 and all tasks in L2) were in the recognition of anger.

Overall, CG’s basic prosodic skills were almost fully spared in L1, with mild deficits circumscribed to her L2 (Figure 3B).

## Discussion

In the neurolinguistics literature, pragmatic processing has been traditionally associated with RH networks. However, the evidence presented here challenges the oft-quoted assumption that patients with right-sided lesions are necessarily characterized by pragmatic impairments in verbal communication. Despite moderate deficits in L2 prosody, CG did not show deficits in comprehending metaphors and idioms, or understanding indirect speech acts in either of her languages. Also, tests in L1 and L2 evinced her preservation of basic verbal skills, including verbal auditory discrimination, repetition of words and pseudo-words, equivalent recognition, cognate processing, word translation, and grammaticality judgments.

Of note, some of the left-sided regions compromised in CG (left anterior insula, and its underlying white matter, the putamen, and the dorso-lateral amygdala) have been associated with lexical (Klein et al., 1995; Liu et al., 2010; Abutalebi et al., 2013), semantic (Wartenburger et al., 2003), and syntactic (Tschirren et al., 2011; Hernandez et al., 2015) processing in both languages. However, spared lexical and sentence-level skills is not surprising given CG’s preservation of left perisylvian regions, which are crucially implicated in such functions (Vigneau et al., 2006). In the context of the present study, this evidence shows that basic linguistic conditions to understand and perform verbal pragmatic tasks were guaranteed.

In this regard, performance on such pragmatic tasks was considerably spared in the patient despite her extensive RH lesions. CG presented severe damage in the right fronto-insulo-temporal cortices, which have been implicated in processing of figurative language (Martin and McDonald, 2005; Côté et al., 2007) and prosody (Blumstein and Cooper, 1974; Weintraub et al., 1981; Lalande et al., 1992; Dykstra et al., 1995; Ross and Monnot, 2008). Particularly, her RH lesions include the parahippocampal gyrus, which has been linked to judgment correction (Kuperberg et al., 2000); the amygdala, associated with irony perception and emotional processing (Rymarczyk and Grabowska, 2007; Akimoto et al., 2014); the mid and superior temporal gyri, involved in the processing of metaphors (Kircher et al., 2001); the supramarginal and angular gyri, linked to pragmatic integration (Catani and Bambini, 2014); the inferior parietal lobule, involved in theory of mind (Decety and Lamm, 2007); the complete insula, associated with metaphor processing and feeling of social emotions (Mashal et al., 2005; Williams and Bargh, 2008); a portion of the putamen, implicated in the use of formulaic expressions (Sidtis et al., 2009); and the inferior frontal operculum, related to metaphorical feelings (Lacey et al., 2012).

However, CG’s pragmatic skills were notably more preserved than traditional neurocognitive models would propose (Kaplan et al., 1990; Lindell, 2006; Champagne-Lavau and Joanette, 2009). In particular, whereas neurolinguistic theories of bilingualism have proposed pragmatic functions to be putatively rooted in the RH (Fabbro, 1999; Paradis, 2004, 2009), such a hypothesis clashes against CG’s near optimal performance in multiple pragmatic tests in both languages.

One could surmise that CG’s lateralization of functions was perhaps reversed. Whereas most of the population exhibits left-dominance for basic linguistic functions and right-dominance for pragmatics, the remaining percentage exhibit an opposite (or partially opposite) pattern (Segalowitz, 2014). If CG fell in that population, then her preservation of pragmatic skills would be easily explained by models which characterize pragmatics as an asymmetrically lateralized domain. However, if that were the case, then CG should not have complete sparing of basic language functions, as relevant perisylvian areas in the RH were severely damaged. Moreover, CG was right-handed, which more strongly suggests left-dominance for basic linguistic functions. Also, as seen in tasks tapping emotional prosody skills (mostly linked with the amygdala and insula), and as further shown in previously reported assessments of sensory perception and emotional arousal (García et al., 2017), the patient *did* exhibit some of the expected impairments following damage to critical brain regions. Therefore, although our findings do not fully exclude other interpretations, the patient’s pragmatic profile could hardly be exclusively accounted for in terms of a deviation from normal neurocognitive organization.

Although CG’s neurocognitive profile may be atypical in several respects (García et al., 2017), the less-than-critical links observed between pragmatics and the RH in her case actually align with abundant neuroscientific findings. Specifically, imaging evidence from healthy subjects has indicated that, apart from the typical RH regions, pragmatic processing also engages classical language areas in the LH (inferior frontal gyrus, superior, middle, inferior temporal gyri, and angular gyrus) (Caplan and Dapretto, 2001; Stemmer, 2008). Indeed, bilateral activation has been found in prosody processing (Kotz et al., 2003) and figurative language (Bottini et al., 1994).

A more plausible explanation of CG’s spared pragmatic processing is that uncompromised LH hubs of bilateral functional networks sufficed for successful task completion. In this sense, RH patients generally preserve their syntactic processing (Brownell et al., 1992), which can help them to understand a communicative intention (if it is expressed linguistically). However, it would seem unlikely for CG’s broad sparing of pragmatic functions after her two strokes to reflect the role of mere syntactic processes. A more parsimonious, straightforward explanation is that pragmatic processes *per se* depend on critical contributions from unaffected LH regions.

Complementarily, it is likely that CG’s spared pragmatic performance partially reflected a strategic reliance on her well-preserved prefrontal networks, implicated in executive functions. CG has already been shown to be unimpaired in attention, numerical, verbal and spatial working memory, and verbal inhibitory control, among other functions (García et al., 2017). In this respect, note that, after stroke, bilinguals generally perform better than monolinguals in the very tasks used to asses these functions in CG, and they typically present less severe signs of aphasia (Paplikar et al., 2018). Moreover, it is conceivable that RH damaged patients may rely more heavily on cognitive control functions (e.g., selective attention, mental flexibility) to engage in social communication. In this sense, working memory has been linked to the production of inferences (Calvo, 2005) and understanding sarcasm (Martin and McDonald, 2005), while in adults with traumatic brain injury it has been associated with poor pragmatic understanding (McDonald et al., 2006). Likewise, executive dysfunction has been implicated in theory-of-mind deficits (Russell, 1997) and linked to pragmatic impairments across various neurological and psychiatric disorders (Morrison-Stewart et al., 1992; McDonald, 1999; Hashimoto et al., 2004; McDonald et al., 2006; Perkins, 2010). Conceivably, then, CG’s preserved pragmatic skills may have profited from her spared executive profile.

What is more, this possibility is reinforced by her lifelong experience as an L2 user, given that bilingualism seems to entail advantages in executive processing (Bialystok and Craik, 2010), including working memory (Calvo et al., 2016b). CG’s use of two languages throughout most of her life could have boosted her executive skills, which, in turn, could have partially contributed to the sparing of pragmatic functions or the use of compensation strategies. This hypothesis aligns with recent works that suggest executive functioning may account for an improved language outcomes after stroke (Alladi et al., 2015; Paplikar et al., 2018).

An exception to the above possibility can be found in CG’s emotional prosody skills. As shown above, CG’s performance in emotional prosody seems to be impaired in L2 and in the repetition task in L1 (with special emphasis in the processing of anger). In this respect, several studies have demonstrated that emotional content seems to hinder comprehension in RH patients (Bloom et al., 1993). Importantly, the bilateral amygdalae have been associated with emotional meaning in prosodic tasks (Frühholz et al., 2011; Frühholz and Grandjean, 2013). Indeed, functional and structural imaging evidence has linked the amygdala and the insula to prosody (Leitman et al., 2016) and socio-emotional processing (Couto et al., 2013). More specifically, portions of the right amygdala, alongside the bilateral superior temporal sulcus and the basal ganglia, seem to be involved in the processing of anger prosody (Sander et al., 2005; Rymarczyk and Grabowska, 2007). Therefore, CG’s deficits in emotional processing may be related to the broad compromise of some of these structures and possibly their connectivity with surrounding regions, particularly including bilateral insular lesions. However, while subcortical structures have been distinctively linked to prosody processing in male samples, women seem to rely more on prefrontal regions (anterior cortex) (Rymarczyk and Grabowska, 2007). While this may represent another factor partially underlying the patient’s outcomes, the possible role of gender as a modulator of pragmatic performance after brain damage is not yet well understood and should be further assessed in future research.

Finally, it is worth noting that our patient had a significantly lower AoA than the controls. This is relevant because AoA has been found to impact on bilingual performance (Perani et al., 2003; Bialystok, 2006; Birdsong, 2006), and it might seem to explain the patient’s preserved outcomes in L2 tasks. Yet, all reported results were adjusted for AoA, showing that patterns of spared skills were uninfluenced by this factor. Moreover, AoA could hardly account for the optimal results found in the L1 tasks. Thus, it would seem unlikely for AoA to explain CG’s well preserved linguistic and pragmatic skills in both languages. However, future studies evaluating AoA in brain damage patients will be required to shed light on our hypothesis.

Importantly, the high (and sometimes optimal) results observed in our study were obtained through highly sensitive instruments. The BAT (Paradis, 1987) is the most widely used battery to test bilinguals after stroke and other neurological disorders (Fabbro, 2001; Zanini et al., 2004; Lorenzen and Murray, 2008; Tavano et al., 2008; Faroqi-Shah et al., 2010; Gomez-Ruiz and Aguilar-Alonso, 2011; Nardone et al., 2011; Paradis and Libben, 2014; Paplikar et al., 2018). In addition, it has been rigorously adapted for more than 70 languages (Paradis and Libben, 2014) and has proved to be sensitive to deficits associated with RH damage in patients with different language pair structures and showing different recovery damage (Fabbro and Paradis, 1995; Aglioti et al., 1996; Fabbro et al., 2000; Fabbro, 2001; Abutalebi et al., 2009). In the same vein, the MEC (Ferreres et al., 2007) is a well-known battery for assessment of verbal pragmatics which has yielded valid and reliable results in RH damage patients from same cultural context as CG (Côté et al., 2007; Fonseca et al., 2007, 2008; Abusamra et al., 2009; Ferré et al., 2011) –thus meeting a crucial requisite for the assessment of pragmatics (Gallagher and Prutting, 1983; Lesser and Algar, 1995; Alarcón, 2009). Thus, the patient’s high outcomes in both languages emerged through gold-standard, culturally valid measures of language and pragmatics. Again, however, more studies using instruments from other cultural contexts would be needed to confirm our claims.

In sum, CG’s cognitively preserved profile together with her bilingual experience may contribute to the understanding of pragmatic processing in bilingual patients, in general, and compensation after stroke, in particular. Although the specific mechanisms underlying CG’s pragmatic profile remain debatable, our findings align with previous evidence to suggest that at least some pragmatic functions, in both L1 and L2, may not actually be asymmetrically subserved by the RH. Contrary to what traditional (Bryan, 1989; Kaplan et al., 1990; Lindell, 2006; Champagne-Lavau and Joanette, 2009) and even more recent (Pobric et al., 2008; Parola et al., 2016) works have proposed, it would seem that pragmatic skills depend on widespread networks spanning both hemispheres and including perisylvian and prefrontal regions also involved in basic linguistic mechanisms and executive functions. Our results warrant the conclusion that pragmatic functions may also be subserved by bilateral networks and that, whereas the RH may be more critically related to pragmatics than basic language functions, this does not mean that it constitutes the *putative basis* of the former. Moreover, our findings shed light on the importance of considering pragmatics when assessing cognitive compensation in bilingual patients with right-sided lesions.

### Limitations and Avenues for Further Research

Our work features a number of limitations which could be addressed in further research. First, the patient’s implant prevented us from obtaining functional imaging data. Future studies should employ fMRI methods to examine the relative contribution of RH and LH regions associated with pragmatic processing in relevant lesion models (Stemmer and Whitaker, 2008). Also, the role of each hemisphere during pragmatic processing could be further explored in healthy subjects. Such investigations could also profit from brain connectivity measures to explore the coupling and decoupling of critical hubs across hemispheres (Grefkes and Fink, 2011; Tomasi and Volkow, 2011).

Second, evidence from single case studies may not be easily generalizable (Hartley, 2004). Here, in particular, the distinctive linguistic and executive profile of bilingual subjects suggests that different pragmatic patterns might be observed in monolinguals (Bialystok et al., 2009). However, case studies have historically led to pioneering advances in the study of language organization (Broca, 1861; see also Ryalls and Lecours, 1996), especially when linguistic functions are preserved in abnormal brains (see Piattelli-Palmarini, 2017). Importantly, our report may be directly relevant for bilingualism research: as our patient does not show cognitive or linguistic impairment after two strokes, her outcomes may reflect compensatory effects associated with high cognitive reserve, thus potentially informing a thriving area of inquiry (Bialystok et al., 2007; Cabeza et al., 2018). Moreover, lesion studies in patients with enhanced executive processing (e.g., bilinguals, music experts) can be useful to investigate the relationship between pragmatic processing and cognitive compensation.

## Conclusion

Throughout the history of neuropsychology, single cases have been paramount to understand the organization of diverse cognitive functions in the human brain (Macmillan, 2000; Corkin, 2002; Dronkers et al., 2007). Here, the evidence afforded by CG, alongside several other works discussed above, invites new reflections on the alleged putative role of the RH in pragmatic domains, suggesting that such a relation may have been overemphasized in the literature. Despite its limitations, the case presented here shows a pattern of preserved pragmatic skills in the patients’ two languages despite extensive lesions to RH areas previously proposed to constitute *putative* basis of such functions. In this sense, CG’s case extends previous findings on the distributed neural organization of pragmatic networks, arguing against localizationist views of pragmatic processing, in particular, and cognitive functions, in general.

## Author Contributions

AI, MMC, and AG conceived and designed the study. NC, SA, MMC, BS, and DZ interviewed and evaluated the patient and the controls. SA, LS, and NC analyzed the images. NC performed the statistical analysis of all tests. NC, LS, and AG designed the figures. NC wrote the manuscript. AG, LS, and AI provided critical revisions on successive drafts. All authors approved the manuscript in its final form.

## Conflict of Interest Statement

The authors declare that the research was conducted in the absence of any commercial or financial relationships that could be construed as a potential conflict of interest.
