# Supplementary material for: Rethinking the Neural Basis of Prosody and Non-literal Language: Spared Pragmatics and Cognitive Compensation in a Bilingual With Extensive Right-Hemisphere Damage
Source: Front Psychol. 2019 Mar 19;10:570. doi: 10.3389/fpsyg.2019.00570 (PMC6433823; doi:10.3389/fpsyg.2019.00570)
Supplement: Supplementary file 1 [file Data_Sheet_1.docx]

**Supplementary material**

**Supplementary Table 1.** Extension of damage per region in the patient.

| **AAL Atlas labels (ID)** | **Affected regions** | **Lesion extension (%)** |
| --- | --- | --- |
| FAD (2002) | Precentral gyrus L | 1.62% |
| F2D (2202) | Middle Frontal gyrus R | 9.32% |
| F3OPD (2302) | Inferior frontal operculum R | 24.05% |
| F3TD (2312) | Inferior frontal gyrus, pars triangularis R | 10.23% |
| F3OD (2322) | Orbital part of the inferior frontal gyrus R | 14.91% |
| ORD (2332) | Rolandic operculum R | 28% |
| COBG (2501) | Olfactory cortex L | 10.61% |
| ING (3001) | Insular cortex L | 12.22% |
| IND (3002) | Insular cortex R | 73.12% |
| CIPD (4022) | Posterior cingulum cortex R | 7.54% |
| HIPPOG (4101) | Hippocampus L | 3.94% |
| HIPPOD (4102) | Hippocampus R | 2.84% |
| PARA_HIPPOG (4111) | Parahippocampal gyrus L | 1.50% |
| PARA_HIPPOD (4112) | Parahippocampal gyrus R | 14.03% |
| AMYGDG (4201) | Amygdala L | 69.13% |
| AMYGDD (4202) | Amygdala R | 35.62% |
| V1D (5002) | Calcarine cortex R | 11.17% |
| LINGD (5022) | Lingual gyrus R | 11.78% |
| O1D (5102) | Superior occipital cortex R | 4.58% |
| O2D (5202) | Middle occipital cortex R | 52.88% |
| FUSID (5402) | Fusiform gyrus R | 7.52% |
| PAD (6002) | Postcentral gyrus R | 1.75% |
| P1D (6102) | Superior parietal cortex R | 6.17% |
| P2D (6202) | Inferior parietal cortex R | 66.14% |
| GSMD (6212) | Supramarginal gyrus R | 33.65% |
| GAD (6222) | Angular gyrus R | 72.74% |
| PQD (6302) | Precuneus R | 1.23% |
| NLG (7011) | Putamen L | 39.94% |
| NLD (7012) | Putamen R | 27.11% |
| PALLG (7021) | Globus pallidus L | 5.38% |
| HESCHLG (8101) | Heschl’s gyrus L | 1.50% |
| HESCHLG (8102) | Heschl’s gyrus R | 91.43% |
| T1D (8112) | Superior temporal gyrus R | 80.32% |
| T1AG (8121) | Superior temporal pole L | 3.65% |
| T1AD (8122) | Superior temporal pole R | 34.09% |
| T2D (8202) | Middle temporal gyrus R | 71.82% |
| T2AD (8212) | Middle temporal pole R | 41.61% |
| T3D (8302) | Inferior temporal gyrus R | 32.25% |
| As in previous works of our group (García-Cordero et al., 2016), to estimate the extension of the lesion in each area in the MNI space, we used the Automated Anatomical Labeling (AAL) Atlas (Tzourio-Mazoyer et al., 2002) to parcel the normalized lesion-mask of the patient. Based on this parcellation, we calculated the percent of volume of each AAL region (116 in total) that was covered by the extent of the lesion-mask. We only included the regions that present at least > 1% of lesion according to this method. | | |

**
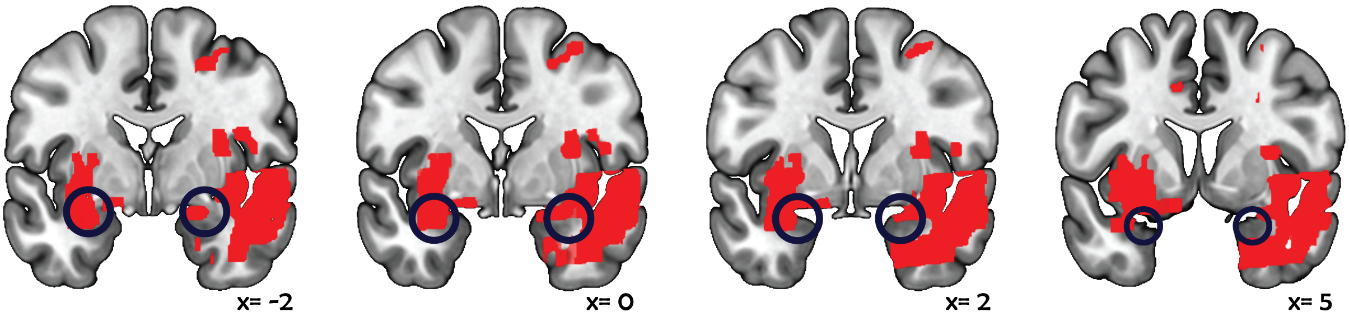
**

**Supplementary Figure 1.** Additional sections highlighting damage to the left and right amygdalae.

**Supplementary references**

García-Cordero, I; Sedeño, L; De la fuente, L; Slachevsky, A; Forno, G; Klein, F; Lillo, P; Ferrari, J; Rodriguez, C; Bustin, J; Torralva, T; Baez, S; Yoris, A; Estevez, S; Melloni, M; Salamone, P; Huepe, D; Manes, F, García, A; Ibáñez, A. (2016). Feeling, learning from and being aware of inner states: interoceptive dimensions in neurodegeneration and stroke. Philosophical Transactions of the Royal Society B: Biological Sciences. 371:1708. doi: 10.1098/rstb.2016.0006

Tzourio-Mazoyer, N., Landeau, B., Papathanassiou, D., Crivello, F., Etard, O., Delcroix, N., Mazoyer, B; Joliot, M. (2002). Automated anatomical labeling of activations in SPM using a macroscopic anatomical parcellation of the MNI MRI single-subject brain. Neuroimage.15:1. doi: :10.1006/nimg.2001.0978
